# Spontaneous Rectus Sheath Hematoma Presenting As Acute Abdomen in an Anticoagulated Patient: A Case Report

**DOI:** 10.7759/cureus.109486

**Published:** 2026-05-23

**Authors:** Ali K Hasan, Sakina K Hasan, Najla M Alsudairy

**Affiliations:** 1 General Practice, Salmaniya Medical Complex, Manama, BHR; 2 Anesthesia, Salmaniya Medical Complex, Manama, BHR; 3 Family Medicine, Ministry of National Guard Health Affairs, Jeddah, SAU

**Keywords:** abdominal pain, acute abdomen, anticoagulation, case report, computed tomography, conservative management, inferior epigastric artery, rectus abdominis hematoma, rectus sheath hematoma, warfarin

## Abstract

Rectus sheath hematoma is an uncommon but important cause of acute abdominal pain that may closely mimic intra-abdominal surgical emergencies, resulting in diagnostic uncertainty and potential delays in management. We report the case of a 64-year-old woman receiving warfarin therapy for chronic atrial fibrillation who presented with sudden-onset severe lower abdominal pain associated with abdominal distension and signs suggestive of an acute abdomen. Clinical evaluation revealed localized abdominal wall tenderness, a palpable infraumbilical mass, anemia, and supratherapeutic anticoagulation. Contrast-enhanced computed tomography (CT) demonstrated a large left-sided rectus sheath hematoma extending below the arcuate line into the prevesical space, causing significant mass effect without evidence of active extravasation. The patient was managed successfully with conservative treatment, including discontinuation of anticoagulation, reversal of coagulopathy, blood transfusion, analgesia, and close hemodynamic monitoring, resulting in progressive clinical recovery and significant interval resolution on follow-up imaging. This case highlights the importance of considering rectus sheath hematoma in the differential diagnosis of acute abdomen, particularly in elderly anticoagulated patients or those with recent episodes of increased intra-abdominal pressure. Prompt recognition through careful clinical assessment and early cross-sectional imaging is essential to avoid unnecessary surgical intervention and to facilitate appropriate management.

## Introduction

Rectus sheath hematoma is an uncommon but increasingly recognized cause of acute abdominal pain resulting from bleeding into the rectus abdominis muscle or the surrounding rectus sheath, most commonly due to injury of the superior or inferior epigastric vessels [1,2]. Although traditionally considered a rare clinical entity, its incidence has risen with the widespread use of anticoagulant and antiplatelet therapies, particularly among elderly patients with multiple comorbidities. Additional predisposing factors include trauma, vigorous coughing, abdominal wall strain, hypertension, pregnancy, and previous abdominal surgery [3,4]. The clinical presentation is highly variable and may range from mild localized pain to hemodynamic instability and acute abdomen, frequently mimicking more common intra-abdominal surgical emergencies such as appendicitis, perforated viscus, diverticulitis, or bowel obstruction [1,3]. Consequently, delayed or incorrect diagnosis may result in unnecessary surgical intervention and increased morbidity [2,4].

Radiological imaging plays a central role in establishing the diagnosis and guiding management. Contrast-enhanced computed tomography (CT) is considered the diagnostic modality of choice because of its high sensitivity and ability to determine the size, extent, and presence of active bleeding [1,3,4]. Magnetic resonance imaging may further aid in tissue characterization and exclusion of underlying neoplastic or soft tissue pathology in selected cases [1,3]. Most rectus sheath hematomas can be managed conservatively with analgesia, fluid resuscitation, blood transfusion, and correction of coagulopathy, while invasive intervention is reserved for patients with ongoing hemorrhage or hemodynamic compromise [1-3]. This case highlights the diagnostic challenge posed by rectus abdominis hematoma presenting as acute abdomen and emphasizes the importance of maintaining a high index of suspicion in anticoagulated patients.

## Case presentation

A 64-year-old woman presented to the emergency department with a sudden onset of severe lower abdominal pain of 12 hours’ duration. The pain was initially localized to the left lower quadrant but progressively became diffuse and was associated with abdominal distension, nausea, and difficulty ambulating due to worsening discomfort. She described the pain as sharp, constant, and exacerbated by movement, coughing, and attempts to sit upright. There was no history of trauma, recent surgery, fever, hematemesis, melena, or rectal bleeding. Her medical history was significant for poorly controlled hypertension, chronic atrial fibrillation for which she had been receiving oral anticoagulation therapy with warfarin, and type 2 diabetes mellitus. She denied any previous history of abdominal wall masses or hernias. The patient reported a recent episode of vigorous coughing secondary to an upper respiratory tract infection during the preceding week. Her medication history included warfarin, metoprolol, amlodipine, and metformin. There was no known history of bleeding disorders or previous spontaneous hematoma formation.

On arrival, the patient appeared acutely distressed because of pain. Initial vital signs revealed a blood pressure of 96/62 mmHg, pulse rate of 112 beats/minute with irregular rhythm, respiratory rate of 22 breaths/minute, temperature of 36.8°C, and oxygen saturation of 98% on room air. Physical examination demonstrated marked tenderness over the lower abdomen, predominantly involving the left paraumbilical and infraumbilical regions, with localized guarding and rebound tenderness that clinically mimicked an acute surgical abdomen. A palpable, tender abdominal wall mass measuring approximately 8 × 6 cm was appreciated over the left lower anterior abdominal wall. The pain intensified with contraction of the abdominal musculature during Carnett’s test, suggesting an abdominal wall origin. Ecchymosis was absent on initial presentation. Bowel sounds were preserved, and digital rectal examination was unremarkable. Cardiovascular and respiratory examinations revealed no additional abnormalities except for an irregular pulse consistent with atrial fibrillation.

Initial laboratory investigations demonstrated hemoglobin of 9.8 g/dL compared with a documented baseline value of 12.7 g/dL two months earlier. White blood cell count was 11.6 × 10^9^/L, platelet count was within normal limits, and serum lactate was mildly elevated at 2.3 mmol/L. Coagulation studies revealed a markedly prolonged international normalized ratio (INR) of 4.1. Renal and liver function tests were within normal ranges. Serum amylase, lipase, and cardiac biomarkers were unremarkable. Urinalysis did not demonstrate hematuria or evidence of urinary tract infection. Given the acute abdominal presentation and hemodynamic instability, urgent radiological evaluation was pursued to exclude intra-abdominal catastrophe.

Abdominal ultrasound revealed a heterogeneously hypoechoic area within the anterior abdominal wall (Figure 1). Contrast-enhanced CT of the abdomen and pelvis demonstrated a large, heterogeneous, hyperdense collection within the rectus abdominis muscle, extending inferiorly below the arcuate line into the prevesical space. The lesion measured approximately 11.2 × 6.4 × 14.1 cm. Surrounding inflammatory fat stranding and compression of adjacent bowel loops were noted without evidence of bowel perforation or visceral injury. No active contrast extravasation was identified on the portal venous phase images. The findings were consistent with a large rectus sheath hematoma, likely resulting from injury to the inferior epigastric vessels. The hematoma caused a significant mass effect, explaining the patient’s acute abdominal presentation (Figure 2).

**Figure 1:**
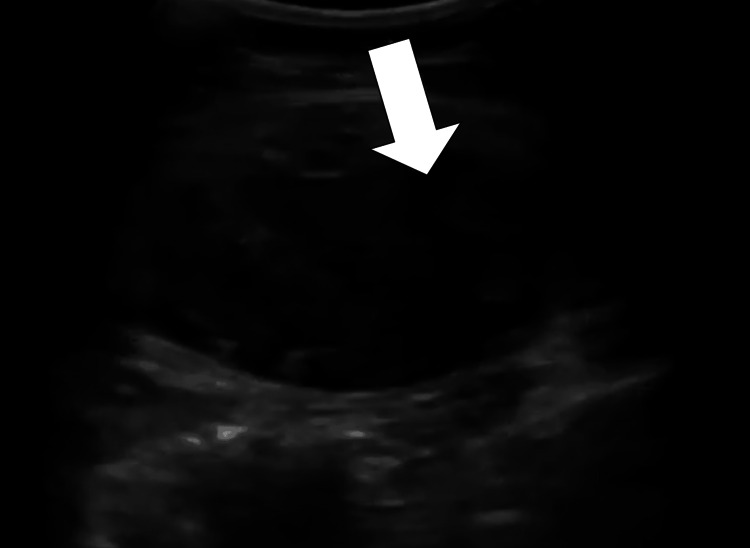
Ultrasound image demonstrating rectus sheath hematoma Gray-scale abdominal ultrasound image demonstrating a heterogeneous hypoechoic collection (arrow) within the left rectus abdominis muscle, consistent with rectus sheath hematoma. No evidence of intra-abdominal free fluid was identified on ultrasonographic examination.

**Figure 2:**
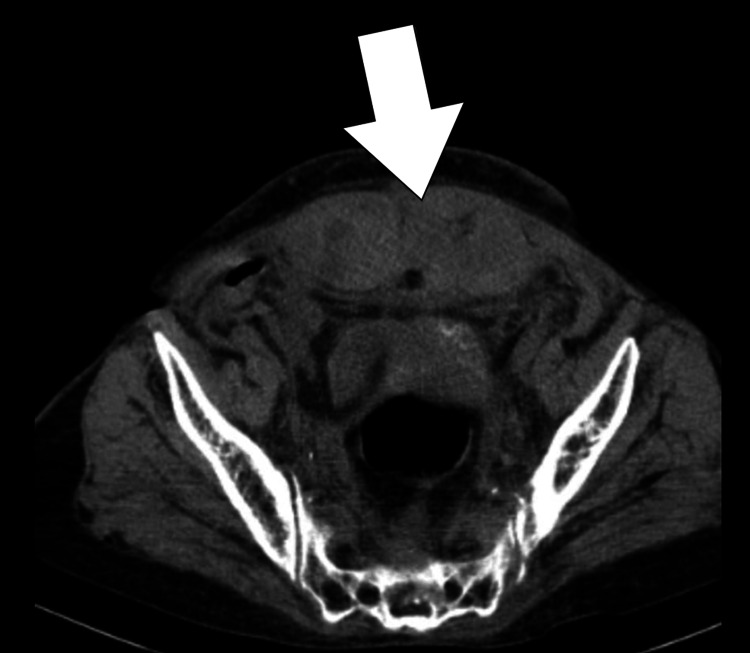
Computed tomography image of rectus sheath hematoma extending into the prevesical space Contrast-enhanced axial computed tomography image of the abdomen and pelvis demonstrating a large heterogeneous hyperdense collection (arrow) within the rectus abdominis muscle, consistent with rectus sheath hematoma. The hematoma extends inferiorly below the arcuate line into the prevesical space, causing mass effect on adjacent bowel loops without evidence of bowel perforation or active contrast extravasation. Surrounding inflammatory fat stranding is also noted.

The patient was admitted to the high-dependency unit for close monitoring and conservative management. Anticoagulation therapy was immediately discontinued, and reversal of coagulopathy was initiated using intravenous vitamin K and fresh frozen plasma. Intravenous crystalloids and opioid analgesia were administered, and serial hemoglobin measurements were performed every six hours. Two units of packed red blood cells were transfused following a further decline in hemoglobin to 8.1 g/dL during the first 24 hours of hospitalization. Surgical consultation recommended non-operative management because the patient remained responsive to resuscitative measures and imaging demonstrated no evidence of ongoing active hemorrhage. Interventional radiology embolization was considered but deferred due to subsequent hemodynamic stabilization and absence of radiological progression.

Over the following days, the patient demonstrated gradual clinical improvement with a reduction in abdominal pain and stabilization of hemoglobin levels. Mobilization was progressively resumed with physiotherapy support. The patient was discharged after eight days of hospitalization in stable condition with instructions for close outpatient follow-up and delayed reintroduction of anticoagulation therapy under cardiology supervision. At the six-week follow-up, the patient remained clinically well with near-complete resolution of symptoms. Follow-up imaging revealed a significant interval reduction in hematoma size without residual complications.

## Discussion

Rectus sheath hematoma represents an uncommon but clinically significant cause of acute abdominal pain that continues to pose substantial diagnostic and therapeutic challenges, particularly in elderly patients receiving anticoagulation therapy [3-5]. The present case illustrates several important aspects of this condition, including its ability to mimic an acute surgical abdomen, the critical role of cross-sectional imaging in establishing the diagnosis, and the effectiveness of conservative management in appropriately selected patients. Despite increasing recognition in modern clinical practice, rectus sheath hematoma remains frequently underdiagnosed because its presentation overlaps considerably with a wide spectrum of intra-abdominal emergencies [3,5,6]. Failure to recognize this entity may lead to unnecessary exploratory laparotomy, delayed treatment, prolonged hospitalization, and avoidable morbidity.

The pathophysiology of rectus sheath hematoma involves rupture of the epigastric vessels or tearing of rectus abdominis muscle fibers with subsequent accumulation of blood within the rectus sheath [3-7]. The inferior epigastric artery is more commonly implicated because the posterior rectus sheath is absent below the arcuate line, allowing hematomas in this region to expand more extensively into the prevesical and pelvic spaces [3,6]. In the present case, the hematoma extended inferiorly below the arcuate line, accounting for the severity of pain and the pronounced acute abdomen presentation. The patient’s markedly elevated INR, combined with repetitive episodes of vigorous coughing, likely served as synergistic precipitating factors leading to vascular disruption and hemorrhage [7-12]. This observation is consistent with previously reported evidence identifying anticoagulation therapy, advanced age, hypertension, chronic cough, and sudden increases in intra-abdominal pressure as major risk factors for spontaneous rectus sheath hematoma.

Clinically, rectus sheath hematoma has been described as a “great masquerader” because it may closely resemble appendicitis, perforated viscus, diverticulitis, incarcerated hernia, pancreatitis, bowel ischemia, or gynecological emergencies. Patients commonly present with acute abdominal pain, palpable abdominal wall mass, ecchymosis, nausea, or signs of hypovolemia [3-7]. However, classic findings such as Cullen’s or Grey Turner’s signs are relatively uncommon and often appear late in the disease course. In this patient, the presence of localized abdominal wall tenderness and worsening pain during abdominal muscle contraction suggested an abdominal wall origin. Carnett’s sign remains a valuable bedside maneuver that may help distinguish abdominal wall pathology from intra-abdominal disease; nevertheless, its utility may be limited in severe pain states or in patients with diffuse guarding. Consequently, radiological evaluation remains indispensable in confirming the diagnosis and excluding competing surgical pathologies [3,5,7].

CT is widely regarded as the gold standard imaging modality for rectus sheath hematoma because of its excellent sensitivity and specificity, rapid availability, and ability to characterize hematoma size, location, extension, and active contrast extravasation [1-4]. CT additionally facilitates classification of rectus sheath hematoma severity, which may assist in therapeutic decision-making. Type I hematomas are typically small and intramuscular; type II lesions may dissect between fascial planes, whereas type III hematomas often extend into the peritoneum or prevesical space and may be associated with hemodynamic instability. The present case demonstrated imaging features consistent with a large type III hematoma, including extension below the arcuate line and compression of adjacent structures [3-5]. Importantly, no active contrast extravasation was identified, supporting the decision for conservative rather than invasive management.

Management strategies for rectus sheath hematoma depend primarily on hemodynamic status, hematoma size, ongoing bleeding, and the presence of comorbidities. Contemporary evidence suggests that most cases can be managed successfully with conservative treatment consisting of fluid resuscitation, reversal of anticoagulation, analgesia, transfusion support, and close monitoring [4,5,8]. In the present case, prompt discontinuation of warfarin and correction of coagulopathy were central to achieving hemostatic stabilization. The patient responded favorably to supportive measures without requiring surgical exploration or angiographic embolization. This outcome aligns with growing literature demonstrating that surgery is rarely indicated and may even worsen bleeding due to disruption of tamponade mechanisms within the rectus sheath. Instead, transcatheter arterial embolization has emerged as the preferred minimally invasive option for patients with active arterial extravasation or persistent hemodynamic instability despite aggressive resuscitation.

The prognosis of rectus sheath hematoma is generally favorable when diagnosis is established early; however, mortality rates remain significant in anticoagulated and elderly populations, particularly in cases associated with delayed recognition or severe hemorrhage. Reported complications include abdominal compartment syndrome, hypovolemic shock, infection, renal impairment, and recurrence after reinitiation of anticoagulation therapy [2-5]. Therefore, individualized risk-benefit assessment is essential when determining the timing of anticoagulant resumption in patients with strong thromboembolic indications. In the present patient, delayed reinstitution of anticoagulation under multidisciplinary supervision allowed safe recovery without recurrent bleeding episodes during follow-up [1,3,6].

This case underscores several clinically important lessons. First, rectus sheath hematoma should always be included in the differential diagnosis of acute abdomen, especially in elderly patients receiving anticoagulation therapy or those with recent episodes of coughing or abdominal strain. Second, careful physical examination combined with early cross-sectional imaging can substantially reduce diagnostic uncertainty and prevent unnecessary surgical procedures. Third, conservative management remains highly effective in the majority of hemodynamically stable patients, even in the presence of large hematomas, provided that close monitoring and timely correction of coagulopathy are implemented. Finally, this case highlights the value of MRI as a complementary imaging modality in atypical presentations or when exclusion of underlying pathology is necessary. Increased awareness of this uncommon but potentially life-threatening condition among emergency physicians, radiologists, internists, and surgeons may facilitate earlier recognition, optimize treatment strategies, and ultimately improve patient outcomes.

## Conclusions

Rectus abdominis hematoma is an uncommon yet important differential diagnosis in patients presenting with acute abdomen, particularly among elderly individuals receiving anticoagulation therapy or experiencing conditions associated with increased intra-abdominal pressure, such as severe coughing. This case highlights how a spontaneous rectus sheath hematoma can closely mimic life-threatening intra-abdominal surgical emergencies, potentially leading to delayed diagnosis and unnecessary operative intervention. Early recognition through careful clinical assessment and prompt cross-sectional imaging, especially contrast-enhanced CT supplemented by MRI when needed, is essential for accurate diagnosis and appropriate management. Conservative treatment with reversal of anticoagulation, hemodynamic stabilization, analgesia, and close monitoring can achieve favorable outcomes in most hemodynamically stable patients, even in cases involving large hematomas. The key takeaway from this report is that maintaining a high index of suspicion for rectus sheath hematoma in high-risk patients with acute abdominal pain can facilitate timely diagnosis, avoid unnecessary surgery, and significantly improve patient outcomes.
